# Use of intensive care, intracranial pressure monitoring, and external ventricular drainage devises in patients with bacterial meningitis, a cohort study

**DOI:** 10.1007/s00701-024-06188-7

**Published:** 2024-07-09

**Authors:** Malte M. Tetens, Casper Roed, Jacob Bodilsen, Lars H. Omland, Helene Mens, Anne-Mette Lebech, Niels Obel, Kirsten Møller, Rune Rasmussen, Christian Overgaard-Steensen, Anders Perner, Morten Ziebell, Åse B. Andersen

**Affiliations:** 1 Department of Infectious Diseases, Copenhagen University Hospital - Rigshospitalet, Esther Møllers Vej 6, DK-2100 Copenhagen Ø, Denmark; 2 Department of Pulmonary Medicine and Infectious Diseases, Copenhagen University Hospital, North Zealand, Hillerød, Denmark; 3 Department of Infectious Diseases, Aalborg University Hospital, Aalborg, Denmark; 4 Department of Clinical Medicine, Aalborg University, Aalborg, Denmark; 5 Department of Clinical Medicine, Faculty of Health and Medical Sciences, University of Copenhagen, Copenhagen, Denmark; 6 Department of Neuroanaesthesiology, Copenhagen University Hospital - Rigshospitalet, Copenhagen, Denmark; 7 Department of Neurosurgery, Copenhagen University Hospital - Rigshospitalet, Copenhagen, Denmark; 8 Department of Intensive Care, Copenhagen University Hospital - Rigshospitalet, Copenhagen, Denmark

**Keywords:** Meningitis, Bacterial, Intracranial pressure, Postoperative complications, Neurosurgery

## Abstract

**Background:**

Bacterial meningitis can cause a life-threatening increase in intracranial pressure (ICP). ICP-targeted treatment including an ICP monitoring device and external ventricular drainage (EVD) may improve outcomes but is also associated with the risk of complications. The frequency of use and complications related to ICP monitoring devices and EVDs among patients with bacterial meningitis remain unknown. We aimed to investigate the use of ICP monitoring devices and EVDs in patients with bacterial meningitis including frequency of increased ICP, drainage of cerebrospinal fluid (CSF), and complications associated with the insertion of ICP monitoring and external ventricular drain (EVD) in patients with bacterial meningitis.

**Method:**

In a single-center prospective cohort study (2017–2021), we examined the frequency of use and complications of ICP-monitoring devices and EVDs in adult patients with bacterial meningitis.

**Results:**

We identified 108 patients with bacterial meningitis admitted during the study period. Of these, 60 were admitted to the intensive care unit (ICU), and 47 received an intracranial device (only ICP monitoring device *N* = 16; EVD *N* = 31). An ICP > 20 mmHg was observed in 8 patients at insertion, and in 21 patients (44%) at any time in the ICU. Cerebrospinal fluid (CSF) was drained in 24 cases (51%). Severe complications (intracranial hemorrhage) related to the device occurred in two patients, but one had a relative contraindication to receiving a device.

**Conclusions:**

Approximately half of the patients with bacterial meningitis needed intensive care and 47 had an intracranial device inserted. While some had conservatively correctable ICP, the majority needed CSF drainage. However, two patients experienced serious adverse events related to the device, potentially contributing to death. Our study highlights that the incremental value of ICP measurement and EVD in managing of bacterial meningitis requires further research.

## Introduction

Increased intracranial pressure (ICP), hydrocephalus, hemorrhagic stroke, and subdural empyema are among the most serious complications of bacterial meningitis. Non-surgical and neurosurgical interventions may be considered according to Danish and European guidelines [2, 13, 15, 21]. The non-surgical interventions include analgesics, sedation, elevation of the head of the bed, intravenous hypertonic saline, and mechanical ventilation to PaCO_2_ 4.5–5 kPa [15, 16].

Because sedation and assisted ventilation are part of the management of patients with acute bacterial meningitis and descending consciousness, the clinical options for non-invasive monitoring of cerebral function, as a marker of ICP, are impaired. ICP can be measured as the opening pressure during a lumbar puncture or by continuous monitoring via an intracranially inserted ICP monitoring device [7, 19].

In patients with increased ICP, ICP-targeted treatment including an external ventricular drainage (EVD) device, may improve outcomes [7–9, 14, 18]. Half of patients with intracranial devices had CSF drained, suggesting a beneficial effect [6]. However, the insertion EVD is associated with a risk of intracranial hemorrhage or infection [11, 20, 22], and the overall effect remains unclear [16]. Although much rarer, complications related to insertion of ICP monitoring device have been reported [5, 10]. While selected patients may benefit from intracranial interventions, the European Society of Clinical Microbiology and Infectious Diseases and the NICE guidelines do not recommend routine use of intracranial pressure monitoring and treatment due to insufficient evidence and concerns about harm [2, 21]. In Sweden, 84% of patients with bacterial meningitis admitted to the ICU receive an intracranial monitoring device [18]. However, most previous research on the use of ICP devices is based on data from traumatology.

In this single-center, cohort study, the objective was to investigate the use of EVD and ICP monitors, including the frequency of increased ICP, drainage of cerebrospinal fluid (CSF), and complications related to these devices in patients with bacterial meningitis.

## Methods

We performed a descriptive prospective cohort study to investigate the frequency of surgical insertion of ICP monitor and EVD devices, the frequency of drainage with EVDs, and complications among consecutive ICU patients with bacterial meningitis between January 2017 and May 2021.

### Setting

We included patients with bacterial meningitis admitted to Copenhagen University Hospital—Rigshospitalet which serves as a tertiary center and accepts patients with bacterial meningitis referred from the Capital Region and the Region Zealand in Denmark (total population of 2.6–2.7 million individuals in the study period 2017–2021 [1]). In Denmark, there is no definite criteria for insertion of ICP monitors or EVDs in patients with bacterial meningitis in the recommendations of the national guidelines [13, 15]. Thus, the decision to insert an ICP monitor or/and EVD was made by the attending consultant in infectious diseases, the intensivist, and neurosurgeon in common and adherent to local guidelines. The indications were: Glasgow Coma Scale score (GCS) < 9, CT or MR scan of the brain suggestive of increased ICP or need of sedation, i.e., agitation or seizures.

### Population

We included all patients with acute community acquired bacterial meningitis admitted to the Copenhagen University Hospital – Rigshospitalet, who had a verified diagnosis of bacterial meningitis defined as either: 1) clinical symptoms and signs consistent with bacterial meningitis and > 10 leukocytes × 10^6^/ml in CSF, or 2) presence of bacteria in the CSF detected by PCR or culture. Patients with bacterial meningitis were transferred to the ICU due to either descending GSC, GSC < 9, convulsion, respiratory failure, or circulatory failure.

**Fig. 1:**
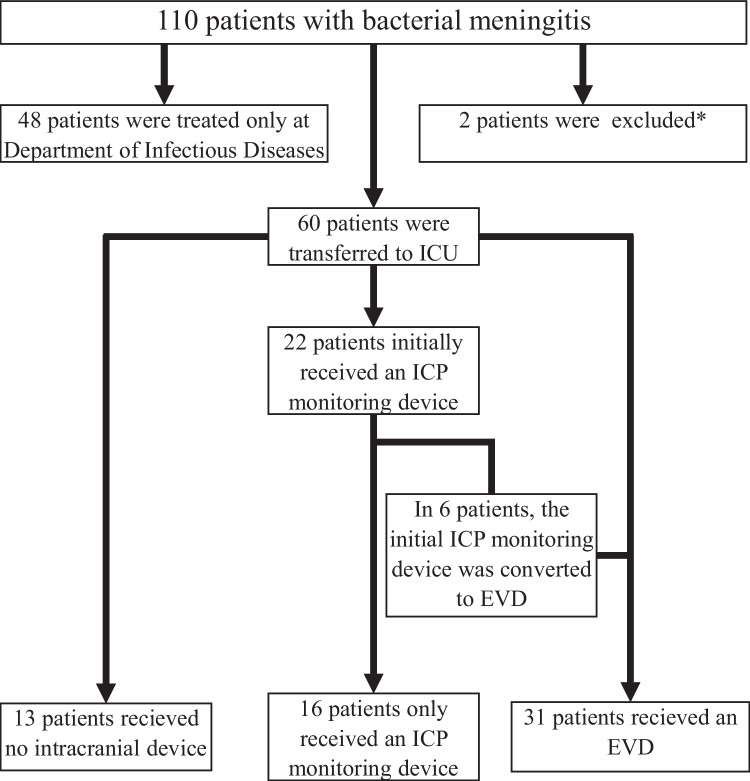
Inclusion of patients admitted to Copenhagen University Hospital – Rigshospitalet with bacterial menigitis between January 1, 2017, and May 1, 2021. *1 case was excluded due to re-admission: First admission without EVD (prior to the study inclusion period) and the second admission with EVD due to hydrocephalus. This patient died. 1 case was excluded because meningitis was caused by *M. tuberculosis*

We excluded individuals with *Mycobacterium tuberculosis* infection and individuals with a previous admission due to bacterial meningitis within one year prior to study inclusion. We categorized patients with bacterial meningitis into three groups (Fig. 1):

1. *Patients without intracranial devices:* Patients with clinical course with no neurosurgical procedures.
2. *Patients with ICP monitoring device:* Patients who received an ICP monitor, but no EVD.
3. *Patients with EVD:* Patients who received an EVD.

**Fig. 2:**
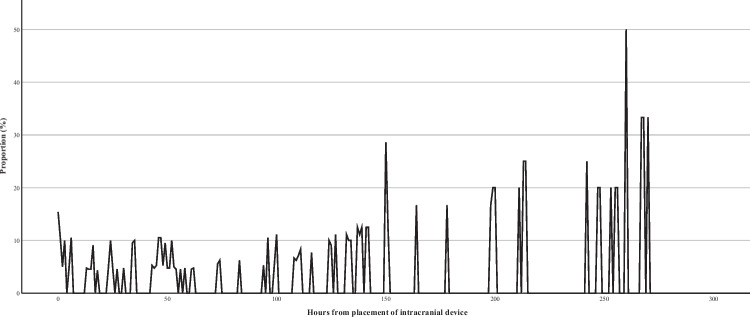
Proportion of patient with intracranial pressure monitor or external ventricular drainage with increased intracranial pressure per hour from implantation of intracranial device

### Data collection and sources

We extracted data on baseline characteristics, duration of symptoms prior to admission, GCS at first admission, bacterial etiology, the use of ICP monitoring and/or EVD devices, non-surgical ICP management, and vital status at 6 months. Data were obtained from the Danish Study Group of Infections of the Brain database [4]. Further, MMT and CR prospectively collected data regarding GCS at admission to Copenhagen University Hospital – Righospitalet, ICU admission, indication for neurosurgical procedure (Glasgow Coma Scale < 9, findings on CT or MRI of the brain suggestive of increased ICP, sedation, convulsion, focal neurological deficit, respiratory or circulatory affection, high lumbar CSF pressure, Cushing response, and status epileptics), use of EVD for drainage, and complications from the patient files (electronically available (Epic, Hyperspace, WI) [17]. In cases of patients with suspected complication related to the neurosurgical intervention, two clinical infectious disease specialists (CR and LO), and a neurosurgeon (RR) assessed the clinical significance of the complication. In cases where the three specialists disagreed, a third clinical infectious diseases specialist (ÅBA) assessed the clinical significance.

### Outcomes

We assessed the use of drainage in patients with intracranial devices, as well as the occurrence of cerebral herniation, intracranial hemorrhage, cerebral abscess, and ventriculitis. Finally, we investigated all-cause mortality at six months.

### Statistical analyses

We used descriptive statistics to calculate the frequency of outcomes.

### Data sharing statement

The ethical approval of this study states the data that has been used in this article cannot be shared publicly.

## Results

Of 108 patients with bacterial meningitis admitted to Copenhagen University Hospital – Rigshospitalet during the study period, 60 were transferred to the ICU and met the inclusion criteria (Fig. 1). Of these, 47 (78%) underwent insertion of either an ICP monitoring device (*n* = 16) or an EVD (*n* = 31). *Streptococcus pneumoniae* was the most common microorganism (Table 1); a higher proportion of patients with an intracranial device had *Streptococcus pneumoniae* as the causative pathogen than those who did not.

**Table 1:** Clinical characteristics of patients with admitted to the ICU at Copenhagen University Hospital – Rigshospitalet (*n* = 60) due to bacterial meningitis between January 2017 and May 2021

	None (*n* = 13)	ICP monitoring device only (*n* = 16)	EVD (*n* = 31)
Age at study admission (years, median, interquartile)	67 (49 – 73)	67 (52 -72)	63 (54–73)
Female sex	5 (38)	10 (64)	12 (39)
Functional status prior to bacterial meningitis			
Retired, totally independent	6 (46.2)	4 (25.0)	10 (32)
Full-time work/study	4 (31)	8 (50)	11 (35)
Disability pension/sick leave or other welfare-based income	1 (8)	1 (6)	3 (10)
Retired, dependent on some help	1 (8)	1 (6)	2 (6)
Unemployed	0 (0.0)	2 (13)	0 (0)
Part-time work/study	0 (0.0)	0 (0.0)	1 (3)
Retired, living in nursing home or equivalent	0 (0.0)	0 (0.0)	1 (3)
Not reported	1 (8)	0 (0)	3 (10)
Immunosuppression			
Diabetes mellitus	2 (15)	1 (6)	7 (23)
Immunosuppressive therapy other than prednisolone	1 (8)	2 (13)	2 (6)
Alcohol abuse	0 (0)	2 (13)	1 (3)
IV substance abuse	0 (0)	1 (6)	0 (0)
Hematological cancer	0 (0)	0 (0)	1 (3)
Asplenia	0 (0)	0 (0)	1 (3)
Prednisolone > 7.5 mg/day	0 (0)	0 (0)	1 (3)
Primary immunodeficiency	0 (0)	0 (0)	1 (3)
Duration of symptoms (days, median, interquartile)	2.5 (1.0 – 4.8)	1.5 (0.3 – 3.0)	1.0 (0.4 – 3.3)
Glasgow coma scale score at admission (median, interquartile)	14 (13 – 15)	11 (7 – 14)	11 (7—14)
Time from admission to empiric meningitis treatment (hours, median, interquartile)	4.4 (1.0 – 8.5)	2.5 (1.2 – 6.9)	3.3 (1.4 – 8.2)
Time from admission to any antibiotic treatment (hours, median, interquartile)	1.6 (1.0 – 5.6)	1.9 (0.7–3.2)	2.6 (1.4–4.2)
Time from admission to lumbar puncture (hours, median, interquartile)	2.8 (1.4–8.2)	1.6 (0.5–6.4)	3.6 (1.1–6.6)
Bacterial etiology			
*Streptococcus pneumoniae*	4 (31)	10 (63)	17 (55)
*Staphylococcus aureus*	4 (31)	3 (18)	0 (0)
*Haemophilus influenzae*	1 (8)	0 (0)	5 (16)
Hemolytic streptococcus	1 (8)	1 (6)	2 (6)
*Neisseria meningitidis*	0 (0)	1 (6)	2 (6)
*Escherichia coli*	0 (0)	0 (0)	1 (3)
Other	1 (8)	1 (6)	3 (10)
Unidentified	2 (15)	0 (0)	1 (3)
Indication for admission to ICU			
Glasgow Coma Score < 9	5 (38)	10 (63)	21 (68)
Convulsions	2 (15)	4 (25.0)	6 (19)
Respiratory failure	9 (69)	8 (50)	12 (39)
Sepsis	7 (54)	7 (44)	10 (32)
Circulatory failure	4 (31)	2 (12.5)	3 (10)
Non-surgical ICP management			
Elevation of the head of the bed	1 (8)	13 (81)	21 (68)
Sedatives	7 (54)	16 (100)	29 (94)
Analgesics	7 (54)	11 (69)	16 (52)
Ventilator set to PaC0_2_ < 4.5–5.0	0 (0)	4 (25)	6 (19)
Hypertonic saline	2 (15)	9 (56)	18 (58)
Indications for intracranial device			
Glasgow Coma Score < 9	6 (46)	8 (50)	22 (71)
Findings on CT of the brain	0 (0)	1 (6)	9 (29)
Findings on MRI of the brain	0 (0)	0 (0)	2 (6)
Sedation	7 (54)	16 (100)	29 (94)
Convulsion	5 (38)	4 (25)	9 (29)
Focal neurological deficit	2 (15)	4 (25)	6 (19)
Respiratory/circulatory affection	9 (69)	6 (38)	13 (42)
High lumbar cerebrospinal fluid pressure	0 (0)	0 (0)	2 (6)
Cushing response^1^	1 (8)	1 (6)	0 (0)
Status epilepticus	0 (0)	0 (0.0)	0 (0)

Increased systolic pressure, bradycardia, and respiratory irregularity

The three groups of ICU patients with bacterial meningitis were similar regarding age, sex, functional level prior to study inclusion, or pre-existing conditions associated with immunosuppression (Table 1). Patients with an intracranial device had slightly shorter symptom duration prior to admission, a slightly lower GCS at first admission, lower GCS at arrival at Copenhagen University Hospital – Rigshospitalet, and shorter time from first admission to administration of empirical antibiotics for meningitis. However, the time from first admission to any antibiotic treatment was not different between the groups. Lumbar puncture was done sooner after first admission in patients with ICP monitoring device compared to those who did not receive an intracranial device, and those who received an EVD.

In general, patients without intracranial devices were admitted to the ICU due to respiratory failure, sepsis, or circulatory failure, whereas patients with an intracranial device were admitted because of severe central nervous system failure such as a GCS < 9 or convulsions. Also, patients with an intracranial device were treated with non-surgical ICP management strategies more frequently while it was rarer in patients without intracranial devices.

Of patients without intracranial devices, more than half fulfilled at least one criterion for receiving an intracranial device, and only one patient without intracranial device fulfilled a recognized contraindication for surgery (disseminated intravascular coagulation). CT- or MR-based signs of increased ICP, i.e., loss of grey matter differentiation, brain herniation, or effacement of the ventricles or basal cisterns, were recorded more often in patients with EVD than in patients with ICP monitoring device.

### Intracranial pressure and EVD drainage

Of 47 patients with an intracranial device, an ICP > 20 mmHg occurred at insertion in 8 and at any time in 21 patients during ICU admission (Fig. 2).

Drainage of CSF was done in 24 (77%) of the 31 patients with an EVD.

### Complications

Complications of bacterial meningitis occurred in all three categories of patients (Tables 2 and 3).

**Table 2:** Number (percentage) of complications in ICU patients with bacterial meningitis between January 2017 and May 2021 (*n* = 60)

	Intracranial device		
Complications	None (*n* = 13)	ICP monitor only (*n* = 16)	EVD (*n* = 31)
Additional antibiotics due to direct intracranial device-related complication^1^	n.a	0 (0)	1 (3)
Additional antibiotics due to CNS complication^2^	0 (0)	0 (0)	7 (23)
Ventilator-associated pneumonia	0 (0)	2 (13)	5 (16)
Aspiration pneumonia	0 (0)	1 (6)	1 (3)
Pulmonary embolism	0 (0)	0 (0)	1 (3)
Catheter-related infection	0 (0)	0 (0)	1 (3)

^1^Cerebral abscess in relation to EVD

^2^Cerebral empyema, ventriculitis, cerebral septic emboli, or clinical deterioration

**Table 3:** Number of CT-verified complications (cerebral hemorrhage, cerebral infarct, cerebral herniation, cerebral abscess, cerebral empyema, and ventriculitis) and deaths in ICU patients with bacterial meningitis (*n* = 60)

Outcome	Numbers at risk	Number of events
Cerebral herniation		
No intracranial device	13	0
ICP monitor	16	0
EVD	31	1
Cerebral abscess		
No intracranial device	13	0
ICP monitor	16	0
EVD	31	1
Cerebral empyema		
No intracranial device	13	1
ICP monitor	16	0
EVD	31	0
Ventriculitis		
No intracranial device	13	0
ICP monitor	16	1
EVD	31	3
Cerebral hemorrhage		
No intracranial device	13	1
ICP monitor	16	5
EVD	31	8
Clinically important cerebral hemorrhage		
No intracranial device	13	0
ICP monitor	16	1
EVD	31	1
Death		
No intracranial device	13	2
ICP monitor	16	2
EVD	31	7

Only nine patients with ICP-monitor or EVD had ventilator-associated pneumonia or aspiration pneumonia.

### Complications with association to neurosurgical procedure

One patient with pneumococcal meningitis and an EVD developed bifrontal cerebritis adjacent to the EVD. Six days after EVD insertion, the patient became febrile, with an increase in the serum leukocyte count increased to 11.0 × 10^9^L (from 5.6 × 10^9^L the previous day), and the first signs of cerebritis were identified on CT-scan. After near normalization of C-reactive protein levels, the C-reactive protein increased again 8 days post-EVD insertion. *Staphylococcus epidermidis* was cultured from CSF drained from the EVD 13 days after the insertion. The patient recieved additional antibiotics (initially vancomycin followed by meropenem) and showed clinically and paraclinically improvement.

CT-verified intracranial hemorrhage occurred in 13 of 47 patients with and in one of 13 patients without intracranial devices (Table 3).

Two patients, both with intracranial devices, sustained fatal intracranial hemorrhage. The intracranial hemorrhage was determined to directly cause the death of one patient and to have contributed to the death of the other. The intracranial hemorrhages were diagnosed on brain imaging performed one and two days after the insertion of the intracranial device. Both hemorrhages were located in close proximity to the intracranial devices. The first hemorrhage, measuring 55 mm × 58 mm × 46 mm, was located in the frontal lope and extended into the ventricles. The second hemorrhage, measuring 33 mm × 31 mm × 41 mm, was located in the frontal lope with extension into the ventricles. One of these patients had a relative contraindication to receive an intracranial device, which may have contributed to the development of the hemorrhage.

### Six-month mortality

The 6-month mortality was 11/60 (18%) overall, 2/13 (15%) in patients without intracranial devices, 2/16 (13%) in patient with ICP monitor, and 7/31 (23%) in patients with EVD.

## Discussion

In this single-center, cohort study of consecutive patients with bacterial meningitis, half of the patients deteriorated clinically and required intensive care. When sedated and intubated the majority had an intracranial device inserted. Half of those patient with an intracranial device had increased ICP at some point of time and needed CSF drainage. One patient with ICP monitoring device died due to intracranial hemorrhage caused by the device, while another patient with an EVD may have died due to intracranial hemorrhage caused by the device. One of these patients had a relative contraindication to receive an intracranial device, which may have contributed to the development of a hemorrhage.

More than half of patients without intracranial devices met at least one indication for insertion of an ICP monitoring device or EVD, and only one patient had a clear contraindication. However, this group of patients presented with a different clinical profile, characterized by more severe systemic organ failure and less severe central nervous system failure, which may have influenced the decision not to insert an intracranial device.

We demonstrated that approximately a third of patients who received an EVD had findings on CT or MRI suggestive of increased ICP. Presence of these signs could indicate that the increased ICP was severe, suggesting that the condition was recognized late and the EVD was inserted too late.

Previous studies have reported varying occurrence rates of increased ICP in ICU patients with bacterial meningitis, ranging between 22 and 87% [6, 8, 18]. This variability may stem from differences in age, sex, microbial etiology, clinical presentation, or small sample sizes. Consistent with our findings, another study group noted that half of patients with intracranial devices underwent CSF drainage [6]. Among patients with bacterial meningitis with an ICP-monitor, 36% to 46% underwent CSF drainage [12, 18], a rate considerably lower than what we observed among patients with EVD, where approximately three out four patients underwent CSF drainage. This disparity likely reflects the selection of patients with the highest ICP for EVD placement rather than ICP-monitoring alone.

Notably, we also observed persistent increased ICP in patients even several days after the insertion of the intracranial device. Access to monitor the ICP may facilitated timely treatment of increasing ICP, potentially benefiting the patient over an extended period. Consistent with these findings, previous studies indicated that the most severely affected patients may derive benefit from ICP monitoring [7]. However, further research seems to be needed to establish a clearer clinical guideline for determining which patients would benefit most from these devices.

Despite frequent use of non-surgical interventions for increased ICP [15, 16], nearly a third of patients initially only monitored with an ICP monitoring device eventually required an EVD. This suggests that non-surgical interventions alone may not adequately control increased ICP in some patients.

Finally, previous studies of EVD treatment for various intracranial disorders have reported risks of infection of 4.8%-5.6% and of hemorrhage of 7%-11%, which is in agreement with our results [11, 22].

The EVD devices used in the study period were capable of monitoring ICP. Therefore, these EVDs may have been inserted in patients with a high clinical suspicion of increased ICP without prior insertion of an ICP monitoring device. This could explain why a quarter of patients with EVD did undergo CSF drainage during their admission in this study.

Wettervik, et al., found an association between poor outcomes and increased ICP among patients with bacterial meningitis [18]. Conversely, Larsen et al., found no association between high ICP and adverse outcomes [12]. Studies by Grände, et al., Lindvall, et al., and Wettervik, et al., indicated that ICP monitoring devices may help guide ICP targeted therapy, but lacked a comparison cohort to investigate overall treatment effects [9, 14, 18]. Conversely, a previous observational study of 37 patients with bacterial meningitis suggested that lumbar drainage of CSF was associated with improved outcome, however this study was limited by study design and small sample size, and it did not access the effect of ICP-monitors or EVD [3]. An intervention-control study with 52 patients found reduced mortality associated with ICP-targeted therapy, but included controls retrospectively, which likely introduced bias [8]. Similarly, our observational study design precluded investigation of the overall treatment effect of ICP monitoring devices and EVD on all-cause mortality or other outcomes in patients with bacterial meningitis.

Previously reported mortality rates of patients with bacterial meningitis with ICP-monitors range from 17–35%, slightly higher than observed in our study [9, 14]. Although our study design did not allow us to investigate overall treatment effect, it is noteworthy that half of patients with an intracranial device had increased ICP necessitating CSF drainage, yet only two cases experienced severe adverse outcomes.

Guyot et al., and Dimitriou et al., reported a rate of hemorrhage up to 0.9% in patients with ICP monitors [5, 10], which is a lower rate than what we observed. The discrepancy between these previous results and the results of our study may be attributable to random variability from small sample sizes or differences in risk of hemorrhages in patients with bacterial meningitis and other patients with need of ICP targeted treatment.

Four out of five of patient with bacterial meningitis in the ICU received an intracranial device, suggesting routine use during the study period. While the insertion of intracranial devices may benefit selected patients, guidelines from the European Society for Clinical Microbiology and Infectious Diseases and the NICE guidelines recommend against routine use of these devices for bacterial meningitis [2, 21]. Importantly, all patients with an intracranial device met at least one indication for its insertion.

### Strengths and limitations

The study is limited by the small study size and by its single-center design, potentially reducing generalizability. The observational design of the study is also a limitation as it may introduce confounding by indication regarding which patients received an ICP-monitor or EVD and a selection bias in terms of which patients were referred to the ICU. Therefore, comparison between groups should be interpreted cautiously.

However, the decreasing incidence of bacterial meningitis and the ethical constraints against conducting randomized trials present significant challenges for investigating the risk–benefit of neurosurgical procedures in such trials. Our data thus provides valuable insights in the absence of lager, randomized trials.

## Conclusions

In this prospective cohort of consecutive ICU patients with acute bacterial meningitis, three quarters of patients underwent insertion of either an ICP monitor or EVD. Half of the patients with an ICP-monitor or EVD underwent CSF drainage, indicating a beneficial effect of these procedures. However, a tenth of patients had adverse events possibly related to the surgical interventions, underscoring the importance of careful consideration of potential contraindications and risk factors for complications. Furthermore, the long-term neurological and cognitive outcomes associated with ICP-monitoring and EVDs in patients with bacterial meningitis remains to be studied.

## Data Availability

The ethical approval of this study from the Rigshospitalet states the data that has been used in this article cannot be shared publicly. Upon a reasonable request to the corresponding author the data can be shared and accessed at our institution.

## Declarations

### Reporting checklist

We adhered to the STROBE Statement checklist when we prepared this manuscript.

### Author instructions

We adhered to Neurocritical Care’s instruction for authors when we prepared this manuscript.
